# Efficiency and prioritization of inference-based credit assignment

**DOI:** 10.1016/j.cub.2021.03.091

**Published:** 2021-07-12

**Authors:** Rani Moran, Peter Dayan, Raymond J. Dolan

**Affiliations:** 1 Max Planck UCL Centre for Computational Psychiatry and Ageing Research, University College London, 10-12 Russell Square, London WC1B 5EH, UK; 2 Wellcome Centre for Human Neuroimaging, University College London, London WC1N 3BG, UK; 3 Max Planck Institute for Biological Cybernetics, Max Planck-Ring 8, 72076 Tübingen, Germany; 4 University of Tübingen, 72074 Tübingen, Germany

**Keywords:** cognitive maps, credit-assignment, reinforcement learning, decision making, model-based, model-free, inference, intrinsic value of information, reward, control

## Abstract

Organisms adapt to their environments by learning to approach states that predict rewards and avoid states associated with punishments. Knowledge about the affective value of states often relies on credit assignment (CA), whereby state values are updated on the basis of reward feedback. Remarkably, humans assign credit to states that are not observed but are instead inferred based on a cognitive map that represents structural knowledge of an environment. A pertinent example is authors attempting to infer the identity of anonymous reviewers to assign them credit or blame and, on this basis, inform future referee recommendations. Although inference is cognitively costly, it is unknown how it influences CA or how it is apportioned between hidden and observable states (for example, both anonymous and revealed reviewers). We addressed these questions in a task that provided choices between lotteries where each led to a unique pair of occasionally rewarding outcome states. On some trials, both states were observable (rendering inference nugatory), whereas on others, the identity of one of the states was concealed. Importantly, by exploiting knowledge of choice-state associations, subjects could infer the identity of this hidden state. We show that having to perform inference reduces state-value updates. Strikingly, and in violation of normative theories, this reduction in CA was selective for the observed outcome alone. These findings have implications for the operation of putative cognitive maps.

## Highlights

• Humans assign credit to hidden task states inferred based on a cognitive map
• The necessity to infer task states taxes the efficiency of credit assignment
• Credit assignment is prioritized for hidden over observable task states
• Inference might be driven by intrinsic (beyond instrumental) value of information

## Teaser

Moran et al. study how credit assignment (CA) and cognitive-map-based inference interact in reinforcement learning. They find that CA is taxed when the identities of hidden task states need to be inferred using a cognitive map, and that, despite the cognitive costs that inference entails, latent states are prioritized over observable states in CA.

## Introduction

Cognitive maps^1^ detail the structure of a decision-making environment, including how states, actions, observations, and rewards are linked.^2^ Much research has focused on how cognitive maps are acquired,^3^, ^4^, ^5^, ^6^ how they support a transfer of structural knowledge to novel environments,^7^ how they support goal-directed planning,^8^^,^^9^ and how they are used “offline” to instruct computationally simpler cached or model-free mechanisms.^10^, ^11^, ^12^, ^13^ Here, we address the role for cognitive maps when inferring latent past states and predicting future states. This type of inference is particularly critical when the values of states need updating on the basis of reward/punishment feedback and where credit must be attributed to relevant antecedent states. Such credit assignment (CA) supports efficient adaptation to the environment, allowing organisms to approach states inferred to be rewarding and avoid those deemed to be punitive.

Humans are remarkably adept at making inferences using cognitive maps to guide CA. For example, authors frequently attempt to infer the identity of insightful or hostile reviewers so as to recommend, or debar, them from future evaluations of their work (depending on whether credit or blame was assigned to them during the current reviews). Furthermore, under conditions of state uncertainty (i.e., when states are only partially observable), people retrospectively infer task states guiding a CA toward an inferred, and away from a non-inferred, state.^14^^,^^15^ Finally, when reward feedback is exuberant and delayed, inference based on a cognitive map is needed to ensure causal attribution of rewards to outcome-relevant antecedent actions.^16^

That inference is cognitively costly^17^^,^^18^ motivated the focus of enquiry for the current study. First, we ask whether updating of state values based on reward feedback (i.e., CA) is compromised by cognitive demands of inference. Second, we ask how any reduction in CA is allocated across the states to which credit could be assigned. For instance, if credit needs to be assigned to observable as well as inferred states (e.g., when an author is reviewed by both signed and anonymous referees), we can ask: are the former favored? A preferential assignment to directly evident states seems intuitive given that CA to inferred states entails a more costly inference process. Alternatively, through the very process of inference the latter might be favored, possibly because there is recruitment of greater attentional resources.

We developed a novel variant of our dual-outcome bandit task,^14^, ^15^, ^16^^,^^19^ which allowed us to address questions related to efficiency and prioritization by comparing CA for states that were either observed or hidden, and where the latter could be inferred based on knowledge stored in a cognitive map. Our task dissociates model-based (MB) and model-free (MF) contributions to choice,^8^,^10^^,^^20^, ^21^, ^22^, ^23^, ^24^, ^25^, ^26^ allowing us to examine any differential impact of state inference on CA with respect to each of these putative controllers.

We show that inference, and particularly retrospective inference, taxed CA associated with MB, but not MF, computations. Strikingly, this CA reduction selectively impacted observed, rather than hidden, states. We discuss mechanistic and normative accounts for these results and how they extend the scope of cognitive-map-based processes, including the possibility for intrinsic value of information gained through inference. Our findings highlight a joint influence of inferential costs and priority in the deployment of cognitive maps for CA.

## Results

### Behavioral task

**Figure 1:**
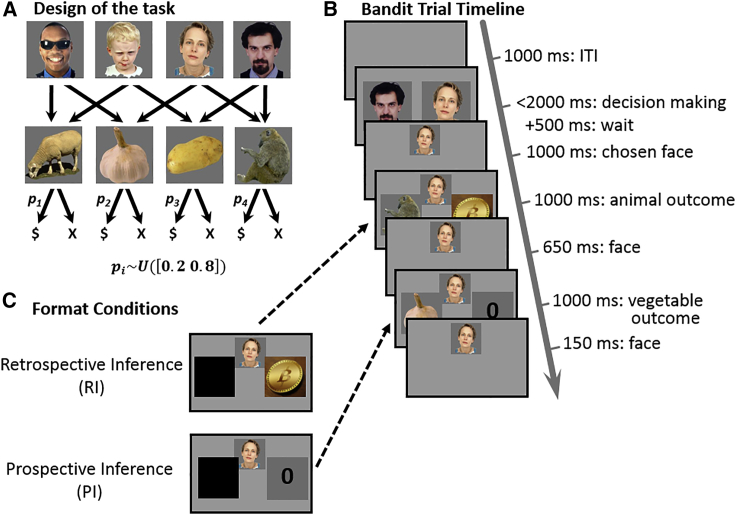
Task structure (A) Participants were introduced to four people, who were each associated with one animal and one vegetable. Each unique vegetable and animal were associated with two people. Animals and vegetables presented as outcomes were probabilistically associated with a coin worth £1. (B) On each trial participants chose, within 2 s, one of two randomly offered people who shared one outcome (animal or vegetable) in common, and subsequently obtained as outcomes this person’s favorite animal and vegetable in random succession. At this point, participants learned for each outcome whether it provided a reward (in the current example trial the monkey yielded a reward, indicated by a coin, but not the garlic, indicated by “0”). Outcomes were presented in one of three formats, randomly interleaved with equal probability. The standard presentation format, wherein the identities of both outcomes were exposed, is illustrated in (B). (C) In the prospective inference format, for the trial illustrated in (B), participants saw a black curtain instead of the second outcome (garlic) but its reward or lack thereof was presented. Importantly, after seeing the first outcome (monkey), participants could predict, based on knowledge of the transition structure (A), the identity of the second outcome. Similarly, in the retrospective inference format, for the trial illustrated in (B), participants saw a curtain instead of the first outcome (monkey). Here, participants could only infer the identity of this first outcome after they saw the second outcome. Note, however, that by that time the reward associated with the hidden outcome was no longer perceptually available. Dashed black arrows indicate the display change necessary to cast the standard trial (B) into an inference format.

In a novel variant of a dual-outcome bandit task,^14^, ^15^, ^16^^,^^19^ we introduced forty participants to pictures of four different people and trained them on the latter’s favorite animal and vegetable. Participants iterated between learning and quiz phases (testing on association knowledge) until they achieved perfect quiz performance (see STAR Methods). By design, each person favored a unique vegetable-animal pair. Crucially, each animal and vegetable were favored by two of these four people (Figure 1A).

Following training, participants played 360 bandit trials (10 blocks x 36 trials) offering a 2-s limited choice between a randomly selected pair of people (Figure 1B). After choosing, participants received as outcome the chosen person’s favorite animal and vegetable in random succession. Each outcome (i.e., animal/vegetable) was associated either with a coin (worth £1) or with nothing (worth £0). On each block of trials, each outcome was governed by a constant reward probability drawn uniformly from the range [0.2, 0.8], independent of other outcomes and across blocks. Subjects were instructed to make choices that maximized earnings.

Crucially, feedback was presented in three different *formats*, randomly interleaved, with equal probabilities. During choice, participants were uninformed about the ensuing presentation format. In a *standard* format (Figure 1B), both outcomes were seen along with their corresponding rewards. In a *prospective* and *retrospective* format, information was withheld during the trial. However, participants were instructed that they could infer the latter from the information available to them. In the prospective format (Figure 1C, bottom), the identity of the first outcome alone was shown, whereas the second outcome was hidden behind a curtain (rewards were presented as normal). Because participants’ model of task structure (i.e., a cognitive map) entailed knowledge of which two outcomes are associated with a chosen person, they could infer the second outcome prospectively, even before it was presented. The retrospective format (Figure 1C, top) was the same as for the prospective format, except now the first outcome was hidden. In this instance, participants could infer what this first outcome was, but could only do so retrospectively (i.e., after seeing the second outcome).

### MFCA and MBCA

We follow a modeling approach described in previous studies.^14^, ^15^, ^16^^,^^19^ This is rooted in dual-systems theory and posits an influence of two distinct information sources during choice^8^^,^^9^^,^^10^^,^^20^, ^21^, ^22^, ^23^, ^24^, ^25^, ^26^, ^27^, ^28^, ^29^, ^30^, ^31^— a rigid, retrospective MF system^32^^,^^33^ and a flexible, prospective MB system.^32^^,^^34^ The MF system repeats actions based upon a past history of action success, whereas the MB system relies on a cognitive map that predicts the impact of actions on both world states and potential future rewards. MF and MB influences are typically integrated during choice.^28^^,^^35^, ^36^, ^37^

**Figure 2:**
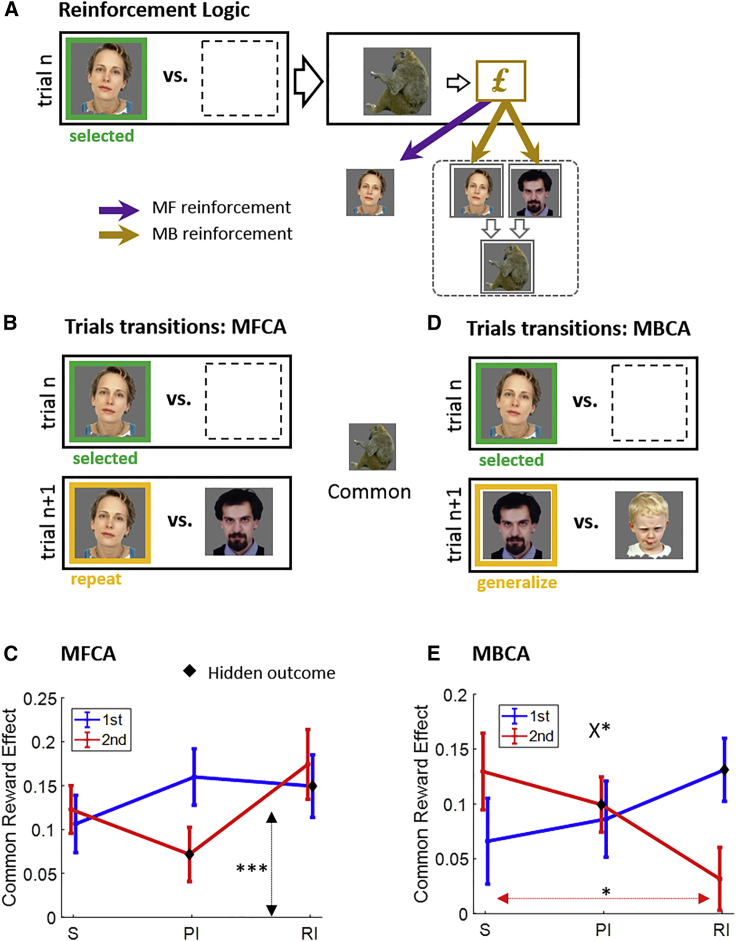
MFCA and MBCA effects (A) After a person (e.g., woman) is selected, an outcome’s (e.g., monkey) reward (or lack thereof) reinforces the MF value of this chosen person alone. In contrast, MBCA will occur for that outcome and, consequently, it will affect equally the values of all people (e.g., woman and bearded man) who share that outcome, because the MB value of each of these people is the sum of the values of his (or her) preferred animal and vegetable. (B) To probe MFCA, we analyzed trials that offered for choice (trial n + 1) the person chosen on the immediately preceding trial n (the repetition person) alongside a person who favored a common outcome, in this example the monkey. We tested the probability of repeating a choice as a function of the previous-trial common reward. The effect of the trial-n common outcome “cancels out” from MB trial n + 1 calculations. Hence, a common reward effect on choice repetition isolates MFCA. For example, when the monkey is rewarded versus not rewarded on trial n, the MF value of the woman will be higher and the probability of repeating the choice will increase. (C) Trial n’s common reward effect on choice repetition (i.e., the difference in repetition probability between common reward and non-reward) is displayed as a function of a trial n’s format (S, standard; PI, prospective inference; RI, retrospective inference) and the common outcome’s serial position on trial n (first/second). The asterisks correspond to the significance of the common reward main effect in the mixed-effects model, corresponding to the average of all 6 data points (vertical black line). (D) To test for an MBCA, we analyzed trials that excluded from choice the person chosen on the immediately preceding trial but offered another person (who we term the generalization person; e.g., the bearded man) who shares an outcome in common (e.g., monkey) with the person chosen previously. Note that the other person offered on trial n + 1 (e.g., the child) shares no outcome with the previously chosen person. (E) Trial n’s common reward effect on choice generalization is displayed as a function of trial n’s format. The asterisks correspond to the significance of the triple interaction (see the X; common reward x format x position) and to the finding that for the second outcome, the common reward effect is stronger in standard versus retrospective inference format (red horizontal line). Error bars correspond to SEM across participants calculated separately in each format. ^∗^p < 0.05, ^∗∗∗^p < 0.001. p values were calculated based on mixed-effects logistic regression models. Black diamonds in (C) and (E) designate concealed outcomes. See Figures S1 and S2 for supporting model simulations and Figure S3 for further analyses.

In our task, an MF system uses the history of rewards associated with each person to estimate a current person value (denoted$\;Q^{\text{MF}}$). Thus, in a trial’s choice phase, retrieved MF values of the two people presented feed into a decision module. In a learning, reward-feedback, phase the MF system updates the $Q^{\text{MF}}$ value of the *chosen* person alone, a process we call model-free credit assignment (MFCA; Figure 2A). Note that an outcome’s identity is irrelevant for MFCA to a chosen person, because both the identity of the chosen person and the ensuing rewards are fully observed. Consequently, we do not predict presentation-format effects on MFCA.

By contrast, at choice, an MB system calculates prospectively on-demand $Q^{\text{MB}}$ values for each person in an offered pair based on the arithmetic sum of the values of the animal and vegetable they prefer:

(Equation 1) $$\;Q^{\text{MB}}\left(\text{person}\right)=Q^{\text{MB}}\left(\text{associated vegetable}\right)+Q^{\text{MB}}\left(\text{associated animal}\right)$$

An MB learner updates the values of the pair of choice outcomes (vegetable and animal) based on reward feedback, such that the value of each outcome increases or decreases depending on whether it was rewarded. We refer to these updates as model-based credit assignment (MBCA). Importantly, unlike MFCA, the MB system generalizes CA across the two people who share a *common* outcome (Figure 2A).

Critically, because MBCA requires knowledge of outcome identity, it requires inference in the prospective and retrospective presentation formats, a dependence likely to incur temporal and cognitive costs.^16^^,^^17^ We hypothesized that these costs would weaken MBCA. Furthermore, there is a fundamental difference between the two inference formats. In the prospective inference format, one can infer the identity of the second, hidden, outcome before it appears, because this is already implied by the first, observed, outcome. By contrast, in the retrospective inference format, the identity of the first, hidden, outcome is uncertain at the point a reward associated with this outcome is presented. Thus, at the same time as assigning credit to the second, observed outcome, the subject has to employ working memory to remember the first reward, and infer what the first outcome must have been. We hypothesized that these extra demands would place a burden on MBCA and, if extra cognitive resources that subjects could recruit were insufficient, then subjects would prioritize MBCA to one or the other outcome.

We examined signatures of MFCA and MBCA to people and outcomes, respectively. We first present “model-agnostic” analyses supported by validating simulations described in full in Figures S1 and S2.

#### No presentation effects on MFCA

We examined whether MFCA contributed to choice and, if so, whether there was a modulation by presentation conditions. Consider (Figure 2B) a focal trial n + 1, which offers for choice the person (e.g., the woman) chosen on the immediately preceding trial n, along with a second person (e.g., the bearded man), with both sharing a common outcome (here, the monkey). If the monkey was rewarded on trial n, then whereas MFCA would credit the woman, rendering her a more likely choice on trial n + 1, MBCA would appreciate that the extra value of the monkey benefits both choices on trial n + 1, and not favor either. Thus, a positive effect of the common outcome’s reward on choice repetition on such trial pairs constitutes a signature of MFCA (Figure S1A).

Here, we examined whether and how this common reward effect varied as a function of presentation format of trial n (standard/prospective inference/retrospective inference) and whether the common outcome came first or second (determining the nature of inference, if any). Using a logistic mixed-effects model (STAR Methods), we found (Figure 2C) a main effect of reward on the common outcome (b = 0.56, F(1,444) = 55.06, p = 6e-13), but none of the higher-order interactions involving the common reward were significant (all p > 0.17). Thus, the chosen person benefits from MFCA, but there is no evidence for modulation by outcome-presentation condition, i.e., format and/or position.

#### Presentation effects on MBCA

Next, we examined the same questions for MBCA. Consider a trial n + 1 that excludes the person chosen on trial n (e.g., the woman; Figure 2D) from the choice set but includes a person (here, the bearded man) who shares a common outcome (the monkey) with the woman. The other person offered on trial n + 1 (here, the child) shares no outcome with the woman. We call a choice of the bearded man on trial n + 1 “generalization” from trial n. To assess MBCA, we examined the effect of a reward to the monkey on generalization, a so-called common reward effect. An MBCA account predicts this common reward effect should be positive because extra value to the monkey benefits the bearded man (and not the child). Here we ask whether this common reward effect depends on trial n’s presentation conditions (format x position; Figure S1B).

A logistic mixed-effects model (Figure 2E; below, “Rn” refers to the n^th^ row in Table 1, which includes statistical tests and interpretations) showed a positive main effect for the common-outcome reward (R1) on choice generalization, meaning greater generalization following a reward (compared to non-reward) to the common outcome. Crucially, evidenced by the crossing of the curves (Figure 2E), choice generalization was subject to a triple-interaction effect (common reward x format x position, R4; there were no lower-order interactions, R2 and R3). Specifically, the common reward effect (on choice generalization) varied as a function of trial n’s format (standard versus retrospective) in different ways for the first and second outcomes (R5–R7). Interpreting this further, we found that the common reward effect for the first outcome was equivalent for the standard and retrospective inference formats (R8), but for the second outcome it was lower in the retrospective inference format (R9). In sum, comparing the standard and retrospective inference formats, the common reward effect on choice generalization was selectively impaired for the second seen outcome (see Figure S3 for further analyses converging on the same conclusions).

**Table 1:** Results and interpretations for our mixed-effects model regressing choice generalization on common reward, format, and serial position

Row	Effect:common reward x	Stats	Interpretation
1	1	b = 0.41, F(1,444) = 28.31, p = 2e-7^a^	positive CRE, i.e., participants generalize their choice more when the common outcome is rewarded versus non-rewarded
2	Format	F(2,444) = 0.3, p = 0.75	no evidence CRE varies as a function of format
3	Position	b = 0.04, F(1,444) = 0.11, p = 0.738	no evidence CRE varies as a function of serial outcome position
4	Position x format	F(2,444) = 3.08, p = 0.047^a^	CRE varies across the three formats in different ways for the first/second outcomes
5	Position x (PI versus S)	b = 0.08, t(444) = 0.29, p = 0.771	
6	Position x (RI versus S)	b = 0.58, t(444) = 2.25, p = 0.025^a^	CRE varies as a function of format (for RI versus S, but not for other format pairs) in different ways for the first and second outcomes
7	Position x (RI versus PI)	b = 0.5, F(1,444) = 3.54, p = 0.061	
8	First outcome: RI versus S	b = 0.18, F(1,444) = 0.95, p = 0.33	for the first outcome, CRE is similar in standard and retrospective inference
9	Second outcome: RI versus S	b = −0.40, F(1,444) = 4.03, p = 0.045^a^	for the second outcome, CRE is lower in retrospective inference than in standard

The first column indexes row numbers. The second column specifies the effect. We are only interested in effects involving the common reward and hence this factor is included in all effects. For example, the name “position” corresponds to the double common reward x position interaction. The third column specifies statistical tests for each effect. The fourth column specifies the interpretation of the finding. CRE, common reward effect.

a Significant effects.

The presentation of the second (seen) outcome in the retrospective inference format enables MBCA for both the seen and the first, inferred outcome. However, the increased difficulty in this condition impairs CA as compared with the standard format, which serves as a neutral baseline against which to interpret the extent of choice generalization. Strikingly, the impairment was confined to the second, observed, outcome alone. MBCA for the first, hidden, outcome was spared. The upshot is that when inference became possible, an MBCA was prioritized for a hidden over seen outcome in the retrospective inference format. Finally, there was no evidence that prospective inference taxed the efficiency of MBCA, presumably because this type of inference is easier.

### Computational modeling

We tested a series of computational models wherein choices were driven by a mixture of MB-MF contributions. In our full model, each outcome was endowed with an MBCA parameter^16^ that quantified the extent outcome value updates (i.e., an increase following a reward and a decrease following non-reward) following reward feedback (STAR Methods), and was free to vary as a joint function of format (standard/prospective inference/retrospective inference) and an outcome’s serial position (first/second; 6 MBCA parameters in total). The model also included a single parameter for MFCA that contributed to MF updates of action values of chosen people.

**Figure 3:**
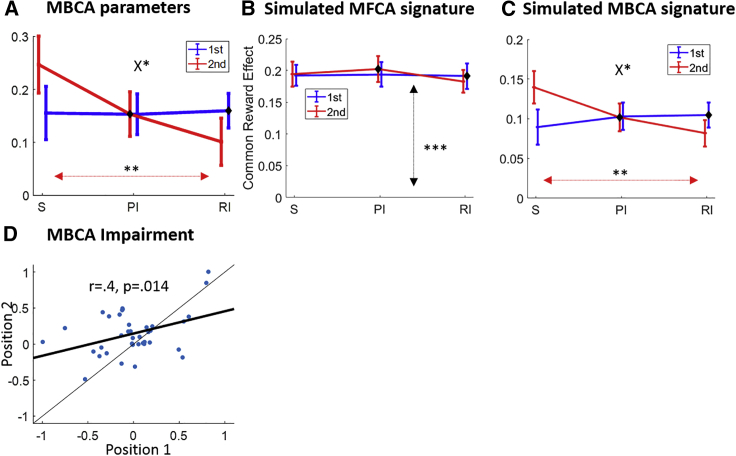
Analysis based on the full computational model (A) MBCA parameters for each format (S, PI, and RI) and outcome serial position (first/second). The asterisks correspond to the significance of the format x position interaction (see the X) and to the standard-retrospective inference contrast for the second outcome (red line). (B) Common-outcome reward effects (MFCA), as in Figure 2C, but based on simulations of the full model. The asterisks correspond to the significance of the main common reward effect. (C) Common-outcome reward effects (MBCA), as in Figure 2E, but based on simulations of the full model. The asterisks correspond to the significance of a triple common reward x format x position (see the X) interaction and to a common reward x standard versus retrospective inference format interaction for the second outcome (red line). (D) MBCA impairment in the retrospective inference format (i.e., the difference between MBCA parameters for the standard and retrospective inference format) for the first (x axis) and second (y axis) outcomes. Each point corresponds to an individual participant. Regression (thick) and identity (thin) lines are imposed. Error bars correspond to SEM across participants calculated separately in each condition. ^∗^p < 0.05, ^∗∗^p < 0.01, ^∗∗∗^p < 0.001. See also Figure S4 for model comparisons, Figure S5 for parameter recovery and trade-offs in the full model, and Table S1 for best-fitting model parameters.

We first conducted ablation studies, comparing our full model to a set of sub-models by lesioning different parts of the full model (STAR Methods). These studies support a conclusion that both MBCA and MFCA processes contribute to choices, and justified an elaborate, interactive, MBCA parameterization in our full model (Figure S4). Additionally, we verified, based on model simulations using participants’ best-fitting parameters, that, unlike the full model, which predicted the model-agnostic signatures presented above (Figures 3B and 3C), each of its sub-models failed to predict a subset of these effects (Figures S1 and S2). Importantly, our full model supported good parameter and model recovery (STAR Methods; Figure S5).

We next probed the best-fitting MBCA parameters in the full model to test how these varied as a joint function of format and position (Figure 3A). A mixed-effects model, wherein we regressed the MBCA parameters on presentation format and outcome serial position (STAR Methods), revealed an interaction between format and position (F(2,222) = 3.10, p = 0.047). Specifically, MBCA was modulated differently by format (standard/retrospective; crossover of curves) for the first and second outcomes (b = 0.15, t(222) = 2.47, p = 0.014; for the other format pairs, both p > 0.135). Interpreting this further, whereas MBCA for the first outcome was on a par across the standard and retrospective inference formats (b = 0, F(1,222) = 0, p = 0.94), MBCA for the second outcome was lower in the retrospective inference format (b = −0.15, F(1,222) = 7.04, p = 0.009). Crucially, these results converge with our model-agnostic analyses to a conclusion that an efficiency cost of retrospective inference was incurred mainly by MBCA for the revealed, as opposed to the hidden, outcome, thereby implicating a prioritization of retrospective inference-based MBCA. Notably, although MBCA for the second outcome was lower in the retrospective inference format, it was still positive (b = 0.1, F(1,222) = 5.38, p = 0.021). We also found that MBCA was positive for the hidden outcome in both inference formats (prospective: b = 0.15, F(1,222) = 13.34, p = 3e-4; retrospective: b = 0.16, F(1,222) = 20.32, p = 1e-5), providing additional evidence that participants relied on a cognitive map of the task to infer the identity of a hidden outcome, fostering MBCA to those outcomes.

Finally, we calculated for each participant the “MBCA impairment” in the retrospective inference relative to the standard format (subtracting a retrospective inference format MBCA parameter from its corresponding standard format parameters), separately for each serial position (Figure 3D). We found a positive across-participants correlation between impairment for the two positions (r = 0.4, p = 0.014), suggesting the difficulty of MBCA in the retrospective inference format is shared by both outcomes. However, the findings also suggest participants respond to this challenge by recruiting additional cognitive resources to ensure MBCA is spared for the hidden outcome. This is evident in a baseline shift for MBCA for the hidden outcome (regressing MBCA impairment for outcome 2 on MBCA for outcome 1 showed a positive intercept b = 0.15, t(36) = 3.27, p = 0.002).

### MBCA is cognitively more demanding in the retrospective inference format

A guiding assumption for our study is that MBCA is more difficult in the retrospective inference condition. We reasoned that if MBCA is more effortful and resource draining in the retrospective inference (compared to standard) format, then choice should be slower on successive trials, at least when this next trial is “new,” i.e., not a replica of the current one (a replica trial offers the same two persons as the previous trial). Because our task includes a long inter-trial interval (Figure 1), which might dilute effort-based inter-trial reaction time (RT) effects, we expected any RT effects to be at best modest.

**Figure 4:**
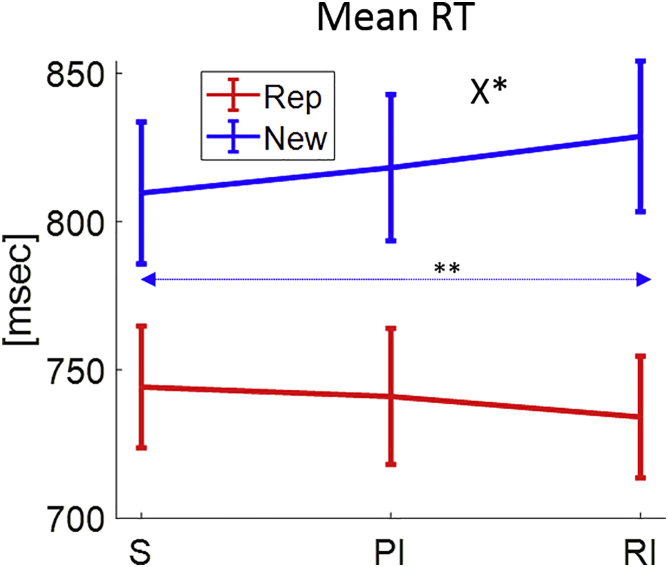
Mean RT results Mean RT is displayed as a function of the previous trial’s format (S, PI, or RI) and the current trial’s type (replica of the previous trial or a new trial). The asterisks correspond to the significance of the format x trial type interaction (see the X) and to the standard-retrospective inference contrast for new trials (blue line). Error bars correspond to SEM across participants calculated separately in each condition. ^∗^p < 0.05, ^∗∗^p < 0.01. p values were calculated based on mixed-effects regression models.

We calculated mean RT for each subject as a function of the previous trial’s format and the current trial type (i.e., replica/new) (Figure 4). A mixed-effects model (STAR Methods) revealed a positive main effect for trial type (b = 79.07, F(1,222) = 63.56, p = 8e-14), showing new trials are slower than replica trials. There was no evidence for a main (previous trial) format effect (F(2,222) = 0.37, p = 0.69). Importantly, however, we found a significant interaction between format and trial type (F(2,222) = 3.27, p = 0.04) such that standard and retrospective inference formats differed dependent on trial type (b = 29.18, t(222) = 2.52, p = 0.012; a prospective inference format did not differ from either standard or retrospective inference formats, both p > 0.229). Furthermore, whereas for replica trials, RT was on a par for the standard and retrospective inference formats (b = −10.09, F(1,222) = 1.26, p = 0.263), for new trials, consistent with our assumption, RT was slower for the retrospective inference format (b = 19.08, F(1,222) = 8.56, p = 0.004).

### Inference-presentation formats tax reward acquisition

**Figure 5:**
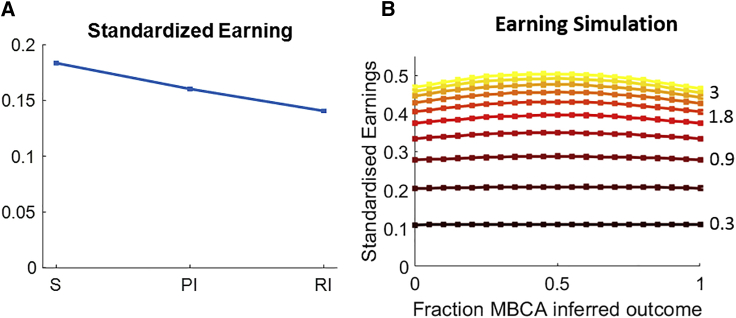
Simulated reward earnings for various regimes of MBCA parameters (A) Average standardized earning for simulated groups of pure-MB agents. A standard agent duplicated the empirical MBCA parameters of its yoked real participant from the standardized format across all three formats. Similarly, prospective- and retrospective inference agents duplicated MBCA from the prospective inference and retrospective inference presentation formats, respectively (see STAR Methods for full details). Error bars correspond to SEM across experiments calculated separately in each format. (B) Standardized earnings (ordinate) of simulated pure-MB agents (STAR Methods) are displayed as a function of (1) the total weight of MBCA in the retrospective inference format summed across observed and inferred outcomes (color gradient, increasing from black to yellow in steps of 0.3, with some values marked on the right side), and (2) the fraction of that total that is allocated to the inferred outcome (x axis). Earnings are maximized when the ratio is 0.5, i.e., when MBCA for the inferred outcome is identical to MBCA for the observed outcome (for none of the curves was there a point significantly higher than the central point). Each data point is based on 10,000 simulations of synthetic experimental sessions (see STAR Methods for full details).

Heretofore, we assessed the cost of inference in terms of reduction in MBCA. However, the ultimate purpose of MBCA is to promote reward acquisition. Thus, we next ascertained the effectiveness for earning reward of participants’ MBCA across the three formats. Because actual earnings in the task reflect the aggregated contribution of MBCA across all three formats as well as of MFCA, we simulated reward earnings for synthetic agents under different settings of MBCA parameters, corresponding to the three different formats (STAR Methods). Figure 5A shows that the effectiveness of our participants’ MBCA was subject to a gradual decline across the standard, prospective inference, and retrospective inference formats.

### Prioritization of inference-based MBCA is non-instrumental for reward acquisition

A recent reinforcement-learning (RL)-based theory proposes that CA should be prioritized for task states that are likely to be encountered more frequently (reflecting a higher “need”) or those where revaluation fosters reward acquisition if one visited these states (reflecting higher “gain”).^13^^,^^38^ In this framework, our findings raise an important question regarding the effects on acquisition of reward for prioritization of MBCA in the retrospective inference format. Because presentation formats are randomized in our task, there is neither increased need nor gain for MBCA to hidden relative to observed states. Therefore, we do not predict such prioritization would be instrumental for reward earnings.

To examine this, we simulated pure-MB agents performing our task over a broad range of MBCA parameters (STAR Methods). Figure 5B displays earnings for different combinations of total (summed across the first and second positions) retrospective inference MBCA parameters (different curves, where warmer color corresponds to a higher total) and for the MBCA ratio between the first, inferred, outcome and the total (x axis). For any level of total MBCA, earnings are optimized when the MBCAs for the first and second outcomes in the retrospective inference formats are equal (the center of the curve). Furthermore, earnings are symmetric across the two outcomes; that is, flipping the MBCA across serial positions did not make a noticeable difference in earnings. These findings show that, as expected, prioritized MBCA to the inferred outcome is not conducive to increased earnings. Furthermore, because inference is cognitively effortful, CA should be prioritized for the seen outcome. A prioritization of inference-based CA is therefore sub-optimal and our findings suggest that either inference is of intrinsic value, or the very process of making inferences leads to an approximate and inefficient outcome.

Finally, we used robust multiple linear regression to examine how participants’ empirical standardized reward earnings varied as a function of the overall level of MBCA (defined as the average of all 6 MFCAs in the full model) and asymmetries in MBCA across serial positions (the absolute difference between MBCA parameters for the first and second serial positions averaged across the three formats). We found a positive effect of overall MBCA level (b = 0.56, t(35) = 4.84, p = 3e-5) and a negative effect for MBCA asymmetry (b = −0.34, t(35) = −2.23, p = 0.032). Thus, although MBCA was beneficial for reward earnings, asymmetries in CA for the first and second outcomes diminished its benefit.

### Addressing alternative accounts

We considered potential alternative explanations for our findings. First, assume that when the first, hidden, outcome is presented on retrospective inference trials, participants assign credit online based on a belief state in relation to both possible outcomes. Then, when the second outcome is seen, CA would occur for the visible outcome alone (as in the standard condition). By itself, this could not account for our main finding of a diminished MBCA for the second outcome in retrospective inference as compared to standard formats. However, a variant might be that a belief state formed during the first outcome loads working memory, thereby diminishing attention paid to the second outcome’s reward (hindering MBCA to the second outcome based on its reward). In contrast, MBCA for the first outcome would be spared because it is held in memory as its reward is presented.

**Figure 6:**
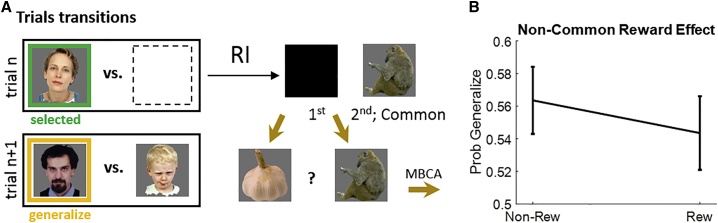
Examining a possibility of cross-credit assignment on retrospective inference trials (A) We reexamined trial transitions of the type used to study MBCA (Figure 2D). Here, however, we considered only retrospective inference format trial n, wherein the common outcome (e.g., monkey; designated as common on trial n + 1) appeared second. If credit from the first reward (related to garlic) is assigned to the second outcome, a reward for the first outcome promotes choice generalization. (B) Choice-generalization probability as a function of the first outcome’s feedback (reward versus non-reward). Following reward, the generalization probability was numerically lower. Error bars correspond to SEM across participants calculated separately in each condition (n = 38). See also Figure S6 for further analyses.

Importantly, this account predicts a form of “cross-credit assignment” such that the value of the second seen outcome is reinforced by a first outcome’s reward. To examine this prediction, we revisited trial transitions of the type used to study MBCA (Figure 2D). Here, however, we considered retrospective inference format trials n alone, wherein the common outcome (e.g., monkey; designated as common on trial n + 1) appeared second (seen). If participants assign credit from the first reward (e.g., related to garlic) to both outcomes, then a garlic reward will reinforce the monkey, promoting choice generalization (Figure 6A). Regressing choice generalization on the first, non-common, reward using a mixed-effects logistic model (STAR Methods), we found a numerically negative effect for the non-common reward (b = −.07, t(74) = −0.58, p = 0.565; Figure 6B). Hence, there was no evidence for online CA based on belief states (see Figure S6 for confirming results based on computational modeling).

Similar considerations speak against the second possibility that what we term MBCA arises due to forms of direct association.^39^ For example, suppose that during outcome, rewards are associated with all persons who relate to a seen outcome. This suggestion, too, cannot account for the diminished MBCA for the second outcome (based on its own feedback) and it is unclear how such associations will account for the MBCA for the first hidden outcome, because throughout the task the black curtain is associated with all 4 persons. Therefore, a reward for the first outcome will be associated with all four persons and will not promote choice generalization. If, however, one assumes that associations for the black curtain occur only for choice-related outcomes, rather than for all four outcomes (an assumption that would itself in any case demand a form of MB inference), then this likewise predicts cross-credit assignment for the first reward. A third potential alternative account for our findings, that the sole effect of the retrospective inference format could be to flip the “functional order” of CA between the two outcomes during feedback, is refuted in Figure S7.

## Discussion

Even when task states are latent, agents can infer these if they hold an internal structural representation of their environment,^40^^,^^41^ i.e., a cognitive map.^1^ Here, we assessed the efficiency costs incurred by a cognitive-map-based guidance of CA, including the relative impact on observed and inferred hidden outcomes.

Because MFCA to actions can operate in our task based on knowledge of actions and rewards alone, we neither expected, nor found, outcome-presentation effects on this process. We previously showed, however, that in other task settings, inferring a hidden state’s identity can guide MFCA toward an inferred state.^14^ Additionally, inference of a latent task state (e.g., which stimulus dimensions predict a reward) can support simpler task representations via dimension reduction and thus aid MF learning.^42^

By contrast, MBCA to individual outcomes (animals and vegetables) is contingent on knowledge of outcome identity, leading us to predict a sensitivity to outcome-presentation format. We show that MBCA occurred for hidden outcomes, consistent with our previous findings that cognitive maps support planning, but also CA.^14^^,^^16^ Notably, the extent to which participants’ MBCA was instrumental to reward earning decreased gradually from the standard to prospective inference and to retrospective inference formats. This pattern is consistent with the idea that the efficacy of MBCA decreases when inference becomes necessary. This reduced efficacy was evident also as an interactive function of presentation format and outcome serial position. Although we found no significant decline in MBCA in the prospective inference format, MBCA was strikingly diminished in the retrospective inference format relative to the standard format for the second, observable, outcome, whereas CA for the first, hidden, outcome was intact.

One potential explanation for why we did not find an expected reduced MBCA in prospective inference, as compared to standard, format trials, is the simplicity of prospecitve inference trials. Future studies might investigate MBCA in more challenging prospective inference setups, for example when the task transition structure entails more complex inferences, or when feedback is delivered under stricter time pressure. Neuroimaging could also be used to test whether a putative anticipatory signal in the prospective inference format predicts the extent of MBCA to the anticipated outcome.

Compared to prospective inference trials, retrospective inference trials impose a greater burden on CA. That non-replicated trials that follow retrospective inference trials have longer RTs is consistent with the extra difficulty. Furthermore, the malign effect of the retrospective inference format on MBCA was correlated across both outcomes (Figure 3D), suggesting this difficulty was shared by both outcomes and that subjects differed in their abilities to mitigate this difficulty. Surprisingly, on average, the difficulty of the retrospective inference condition impacted the seen, as opposed to the hidden, outcome. This suggests that participants recruited additional cognitive resources to ensure CA is uncompromised for the hidden outcome. In other words, our suggestion is that MBCA for the first outcome reflects a balance of two opposing effects, which roughly offset each other. On the one hand, the increased difficulty tends to reduce MBCA for this outcome. On the other hand, there is a positive baseline shift in MBCA for this outcome that we suggest is due to recruitment of additional cognitive resources.

Our computational simulations show that, as might be expected, CA to seen and hidden outcomes is equally instrumental for acquisition of reward (Figure 5B). Furthermore, because we randomized the way we concealed the outcomes, there was equal gain and need in MBCA for seen and hidden outcomes.^13^ Considering the costs of inference and memory maintenance, a normative approach would seem to prioritize a less costly CA to the seen outcome. In contrast, our findings support a conclusion that the more demanding inference-based MBCA was prioritized. This observation points to a novel variable affecting CA prioritization.

Why did participants prioritize MBCA to the hidden outcome in the retrospective inference condition? An algorithmic possibility is that the effort required to infer a hidden outcome directs greater attention to this outcome. This fits with a framework in which information is considered to have intrinsic worth, above and beyond its immediate instrumental value,^43^, ^44^, ^45^, ^46^, ^47^, ^48^ perhaps acquired through prolonged life experiences as a secondary reinforcer. An intrinsic value for information pertaining to a hidden outcome’s identity, which outweighs the cost of inference, could account for this finding.

Finally, our findings have broad implications for ecological decision making given that a need to infer task states is ubiquitous. Our finding suggests that when cognitive resources are limited, CA might be prioritized for inferred states and attenuated for revealed states. We acknowledge our task is idealized in so far as state uncertainty can be fully resolved based on a relatively simple model. In contrast, real life is replete with complex situations wherein inference can only be partially resolved (e.g., inferring the intentions of a close other) based on much richer models (e.g., theory of mind). We think in such situations the extent of CA to task states will be governed by an interplay between the complexity of inference, the resolution it provides, and the available cognitive resources. For example, when resources are scarce, people may adaptively degrade inference, in accordance with bounded optimality accounts.^49^ Relatedly, a potential limitation of our task is that, unlike real-life situations, the duration of the CA phase was under the control of the experimenter. Future studies should examine how inference and CA interact when participants directly control temporal allocation during CA.

In conclusion, an extensive literature highlights both costs and benefits associated with planning based on cognitive maps. The current study provides empirical evidence that cognitive maps play an equally important, and dramatic, role in supporting inference-based CA. Because inference serves a key role in adaptation to natural environments, an important next step will be to characterize with greater granularity the costs and sources of value associated with deployment of cognitive maps.

## STAR★Methods

### Key resources table

**Table:** 

REAGENT or RESOURCE	SOURCE	IDENTIFIER
**Experimental models: organisms/strains**		

Human Participants	NA	NA

**Software and algorithms**		

Matlab ver. (R2020b)	MathWorks	https://www.mathworks.com
Cogent 2000 MATLAB Toolbox	Laboratory of Neurobiology, UCL	https://www.vislab.ucl.ac.uk/cogent.php
Custom scripts for data analyses	Open Science Framework (OSF)	https://osf.io/t3yf9/?view_only=e3e63487443541359b450baaa4d1f593

### Resource availability

#### Lead contact

Further information and requests for resources should be directed to and will be fulfilled by the Lead Contact, Rani Moran (rani.moran@gmail.com)

#### Material availability

This study did not generate new unique reagents.

#### Data and code availability

The data that support the findings of this study and data analysis code have been deposited in the Open Science Framework (OSF) and are available in the following link: https://osf.io/t3yf9/?view_only=e3e63487443541359b450baaa4d1f593

### Experimental models and subject details

#### Participants

Forty participants (15 Males, 25 Females, age range: 20-35) were recruited from the SONA subject pool (https://uclpsychology.sona-systems.com/Default.aspx?ReturnUrl=/) with restrictions of having normal or corrected vision, being non-dyslexic, being a UK based student and being born after 1981. The study was approved by the University College London Research Ethics Committee (Project ID 4446/001). Subjects gave written informed consent before the experiment.

### Method details

#### Experimental procedures

Participants were first familiarised with four pictures of persons and learned which animal-vegetable pair each person grows (pictures of persons, animals, and vegetables were adopted from previous studies^50^^,^^51^). Each vegetable/animal was preferred by two different people, and each person grew a unique animal-vegetable pair. The mapping between persons and animals/vegetables was created randomly anew for each participant and remained stationary throughout the task. After learning, participants were quizzed about which vegetables/animal each person preferred and about which person they would choose to obtain a target animal or vegetable. Participants iterated between learning and quiz phases until they achieved perfect quiz performance (100% accuracy and RT < 3000 ms for each question).

After learning participants played a practice block of 24 trials to verify that they understood the task. These practice trials proceeded as described below with the sole difference that outcome-presentation was only in the standard format. Next, participants were instructed that in following blocks they will either see both choice outcomes or only one choice outcome and the other will be hidden behind a black curtain. They were also instructed that they can infer the identity of a hidden outcome, should they find this useful, based on knowledge of which pair of outcomes they expect to get and the identity of the outcome they saw. Finally, they were instructed that the computer’s decision to hide outcomes and which outcomes to hide is random and independent of their choices.

Participants next played 10 short blocks, each comprising 36 bandit trials. On each trial, a pair from the four persons were offered for choice and participants had 2,000 msec to choose one of these objects (left/right arrow keys). Offered persons always shared one outcome (animal or vegetable) in common. This defined 4 person pairs, each presented on 9 trials (per block), in a random order. Following a choice, participants saw sequentially, and in random order, the two choice outcomes and whether each of these outcomes was rewarded (a notational £1) or not. Importantly, outcomes could be presented in one of three equi-probable formats interleaved across trials. In the standard format both outcomes were seen, in the prospective-inference format only the first outcome was seen and the second was hidden, and in the retrospective-inference format only the second outcome was seen and the first was hidden. Note that rewards were always observable. The reward- probabilities of the four outcomes were constant within each block (four independent uniform samples from [0.2, 0.8]) and independent across blocks.

After each block was completed participants had a forced 40 s (minimum) break. After the break participants were informed that all vegetable/animal reward probabilities were reset to new values and therefore they should form new impressions of these when the task resumes. The task lasted about 60 min. Participants were paid £8 per hour plus a performance-based bonus which was calculated based on the total amount of earned coins on 3 randomly sampled trials (£1 per bonus pt.).

### Quantification and statistical analysis

All statistical analyses were conducted using customary-built scripts and statistical toolbox in MATLAB (R2020b, Natick, Massachusetts: The MathWorks Inc.). All statistical details can be found in the Results and/or figure legends.

#### Participant exclusion

Two participants were excluded, one due to failure to meet the recruitment criteria (not a student) and the other due to not showing any incentive to earn rewards (all 7 CA parameters for this participant were negative). The remaining 38 participants were the targets for the analysis.

#### Model-agnostic analysis: MFCA and MBCA

Our model-agnostic analyses were conducted using logistic mixed effect models (implemented with MATLAB’s function “fitglme”) with participants serving as random effects with a free covariance matrix. In our first analysis (related to Figures 2B and 2C), we used only trials n+1 that offered for choice the trial-n chosen person. Our regressor COMMON coded whether the common outcome was rewarding on trial-n (coded as +0.5 for reward and −0.5 for non-reward), the regressor PROSPECTIVE, and RETROSPECTIVE, indicated whether trial’s n format was prospective-inference or retrospective-inference, respectively, and the regressor POSITION, coded the serial position of the common outcome on trial n (0.5: first, −0.5: second). Variable REPEAT indicated whether the choice on the focal trial n+1 was repeated. PART coded the participant contributing each trial. The model, in Wilkinson notation, was: REPEAT∼COMMON ^∗^(PROSPECTIVE + RETROSPECTIVE)^∗^POSITON + (COMMON ^∗^(PROSPECTIVE + RETROSPECTIVE)^∗^POSITON |PART). We used F-test to examine contrasts on fixed effects. When examining interaction effects involving format (e.g., common-reward x format interaction) we first tested a hypothesis that both the retrospective and retrospective effects (e.g., COMMON ^∗^PROSPECTIVE, COMMON ^∗^ RETROSPECTIVE) were 0 and only if this null was rejected we proceeded to test effects of specific formats (e.g., COMMON ^∗^PROSPECTIVE). Due to format-coding, the main effect for common reward was based on the contrast$\left(3b_{COMMON}+b_{COMMON:PROSPECTIVE}+b_{COMMON:RETROSPECTIVE}\right)/3$. Similarly, the interaction of common-reward x position was based on the contrast$\left(3b_{COMMON:POSITION}+b_{COMMON:POSITION:PROSPECTIVE}+b_{COMMON:POSITION:PROSPECTIVE}\right)/3$.

In our second analysis (related to Figures 2D and 2E), we used only trials n+1 that excluded from choice the trial-n chosen person. Only one of the offered persons (trial n+1) shared a common outcome with the previously chosen person and the variable GENERALIZE indicated whether this person was chosen. We repeated the same analyses as above but for GENERALIZE instead of REPEAT.

Additionally, we ran a mixed effects model (Figure S3A) regressing the common reward effects (the contrast between generalization probabilities following common outcome reward and non-reward) on format and outcome (note each participant contributed 6 effects). The model in Wilkinson’s notation was: REWARD_EFFECT∼POSITION^∗^(PROSEPCTIVE+RETROSPECTIVE)+ (POSITION^∗^PROSEPCTIVE|PART)+(POSITION^∗^RETROSPECTIVE|PART); these regressors are as in previous analyses but here they pertained to a reward effect rather than to single trials.

We also ran a mixed effects logistic regression model, which included all trial transitions and accounted for all the influences that rewards from a previous trial exert via both MBCA and MFCA (Figures S3B and S3C). We regressed the chosen display side (coded in a variable CHOOSE-R/L; left coded as 0, right as 1) on trial n+1 on a set of MBCA and MFCA regressors as follows. First, by task design, it was always the case that exactly one of the two trial-n outcomes (i.e., vegetables/animals), was unique to one of the two persons offered on trial n+1. We label this outcome as the “Unique outcome.” A trial n reward to this Unique outcome supports a choice of the person who is related to that outcome on trial n+1. In contrast, the other trial-n outcome, was either common to both persons offered on trial n+1 or absent from both. Hence, the value of this outcome (and in particular, whether it was rewarded on trial n) exerts no influence on MB choice tendencies on trial n+1. Accordingly, we focused on the unique outcome and we defined the regressor MB_REW as +0.5 if either: 1) the unique outcome relates to the right side person on trial n+1 and was rewarded on trial n, or: 2) the unique outcome relates to the left side person on trial n+1 and was unrewarded on trial n. Otherwise, this regressor was defined as −0.5. Another regressor, POSITION, coded the serial position of the Unique outcome on trial n (−0.5: first, 0.5: second) and the two regressors PROSEPCTIVE and RETROSPECTIVE were defined as in the previous analyses. We next defined a set of MFCA regressors as follows. We had 6 regressors corresponding to the serial position and format of each outcome on trial n. These regressors were named MF_REW1, MF_REW2 (these relate to the standard format), PROSPECTIVE_ MF_REW1, PROSPECTIVE_ MF_REW2 (these relate to the PI format), RETROSPECTIVE_ MF_REW1, RETROSPECTIVE_ MF_REW2 (these relate to the RI format). The numerical 1 or 2 in the regressor’s name corresponds to the serial position of the outcome. If the person chosen on trial n was not offered for choice on trial n+1 then all six regressors were set to 0. Otherwise, the 4 regressors that do not correspond to the trial n format (S/PI/RI) were set to 0. The two regressors corresponding to trial n’s format coded whether the first and second outcome, respectively, were rewarded (0.5) or unrewarded (−0.5) on trial n. However, if the trial n chosen person was on the left side of the screen on trial n+1, we applied a negative sign to all the MFCA regressors. A final regressor, PERS, controlled for choice perseveration tendencies. It was coded as 1, −1 respectively, if the trial n chosen person was offered on the right/left side of the screen on trial n+1, and 0 otherwise. The model, in Wilkinson notation, was: CHOOSE-R/L ∼MB_REW ^∗^(PROSPECTIVE + RETROSPECTIVE)^∗^POSITON + MF_REW1+MF_REW2+PROSPECTIVE_MF_REW1+ PROSPECTIVE_MF_REW2+ RETROSPECTIVE_MF_REW1+ RETROSPECTIVE_MF_REW2+ PERS+ (MB_REW ^∗^(PROSPECTIVE + RETROSPECTIVE)^∗^POSITON + MF_REW1+MF_REW2+PROSPECTIVE_MF_REW1+ PROSPECTIVE_MF_REW2+ RETROSPECTIVE_MF_REW1+ RETROSPECTIVE_MF_REW2+ PERS |PART). We examined fixed effect and contrasts between fixed effects in this model, using F tests as explained above.

Finally, for the “cross credit assignment” analysis (Figure 6B) we included only choice generalization trial-transitions (Figure 6A) such that trial n’s format was RI and the outcome designated as common (on trial n+1) was temporally second (seen) on trial n. Our regressor NON-COMMON coded whether the non-common, first (unseen), outcome was rewarding on trial-n (coded as +0.5 for reward and −0.5 for non-reward). The model, in Wilkinson notation, was: GENERALIZE∼NON-COMMON + (NON-COMMON |PART), where GENERALIZE and PART were defined as above. We reran the same model (Figure S6B) this time including only trial transitions wherein the common outcome was first (hence the non-common outcome was second) on an RI format trial n.

#### Mixed effect analysis for RT

We calculated for each participant mean RT (MRT) as a function of the previous trial’s format and the current trial type, i.e., whether it was a replica of the previous trial or a new trial. We used a mixed effects model to regress mean RT on a set of regressors. We removed trials for which the choice was very fast < 250ms (0.0033 of all trials). We also removed the first trial of each block as these trials followed a break. We defined two regressors: The first, TYPE, coded whether the current trial was a replica of the previous trial (−0.5) or a new (+0.5) trial. The regressors PROSPECTIVE and RETROSPECTIVE were similar to the previous analyses (coding the format of the previous trial). The model, in Wilkinson notation, was: MRT∼TYPE^∗^(PROSPECTIVE + RETROSPECTIVE) + (TYPE^∗^(PROSPECTIVE + RETROSPECTIVE)|PART).

#### Computational models

We formulated a hybrid RL model to account for the series of choices for each participant. In the model, choices are contributed by both the MB and MF systems. The MF system caches a $Q^{\text{MF}}$-value for each person, subsequently retrieved when the person is offered for choice. During learning, following reward-feedback, rewards from the various outcomes are used to update the $Q^{\text{MF}}$-value for the chosen person as follows:

(Equation 2) $$Q^{\text{MF}}\left(\text{chosen person}\right)\leftarrow \;\left(1-f^{MF}\right)*Q^{\text{MF}}\left(\text{chosen person}\right)+c^{MF}*r_{total}$$

where the $c^{MF}$ is a free MFCA parameter, $r_{total}$ is the sum of the animal and vegetable rewards (each coded as 1 for reward or −1 for non-reward) and $f^{MF}$(between 0-1) is a free parameter corresponding to forgetting in the MF system. The MF values of the 3 non-chosen persons were subject to forgetting:

(Equation 3) $$Q^{\text{MF}}\left(\text{non chosen person}\right)\leftarrow \left(1-f^{MF}\right)*Q^{\text{MF}}\left(\text{non chosen person}\right)$$

Unlike MF, the MB system maintains $Q^{\text{MB}}$-values for the four different outcomes (vegetables and animals). During choices the $Q^{\text{MB}}$- value for each offered person is calculated based on the transition structure:

(Equation 4) $$Q^{\text{MB}}\left(\text{person}\right)=Q^{\text{MB}}\left(\text{related vegetable}\right)+Q^{\text{MB}}\left(\text{related animal}\right)$$

Following a choice, the MB system updates the $Q^{\text{MB}}$-values of each of the two choice outcomes based on its own reward:

(Equation 5) $$Q^{\text{MB}}\left(\text{outcome}\right)\leftarrow \;\left(1-f^{MB}\right)*Q^{\text{MB}}\left(\text{outcome}\right)+c_{format,pos}^{MB}*r_{outcome}$$

Where $f^{MB}$ (bet. 0-1) is a free parameter corresponding to forgetting in the MB system, format is the trial’s format (S = Standard, PI = Prospective-Inference, RI = Retrospective Inference), pos=1,2 is the outcome’s serial position and $c_{format,pos}^{MB}$ is a free MBCA parameter corresponding to format and position (6 MBCA parameters). The MB values of the 2 outcomes unrelated to the chosen person were subject to forgetting:

(Equation 6) $$Q^{\text{MB}}\left(\text{unrelated outcome}\right)\leftarrow \left(1-f^{MB}\right)*Q^{\text{MB}}\left(\text{unrelated outcome}\right)$$

Our model additionally included progressive perseveration for chosen persons. After each trial the perseveration values of each of the 4 persons updated according to

(Equation 7) $$PERS\left(\text{person}\right)\leftarrow \left(1-f^{P}\right)*PERS\left(\text{person}\right)+\text{pr∗}1_{\text{person}=\text{chosen}}$$

Where $1_{\text{person}=\text{chosen}}$ is the chosen person indicator, pr is a free perseveration parameter, and $f^{P}$(bet. 0-1) is a free perseveration forgetting parameter.

During choice a net Q value was calculated for each offered person:

(Equation 8) $$Q_{\text{net}}\left(\text{person}\right)=Q^{\text{MB}}\left(\text{person}\right)+Q^{\text{MF}}\left(\text{person}\right)+PERS\left(person\right)$$

The $Q_{\text{net}}$ values for the 2 persons offered for choice are then injected into a softmax choice rule such that the probability to choose an option is:

(Equation 9) $$Prob\left(\text{person}\right)=\frac{e^{Q_{\text{net}}\left(\text{person}\right)}}{e^{\left[Q_{\text{net}}\left(\text{person}\right)+Q_{\text{net}}\left(\text{other person}\right)\right]}}$$

$Q^{\text{MF}}$ and PERS person-values and $Q^{\text{MB}}$ outcome-values where initialized to 0 at the beginning of each experimental block.

We also examined an additional more flexible model, which we term the “RI cross CA” model. This model, was identical to the full model described above with a sole change that in the RI format, each reward was allowed to reinforce the MB value of both choice-related outcomes (rather than just its own outcome). Such reinforcement is expected if in the retrospective-inference format, participants assign credit for the first reward as it is presented and prior to fully resolving the identity of the full outcome. This can happen if participants assign credit for the first reward based a belief state (which allows for the possibility that the second, as yet unseen, outcome generated the first reward), or if after choice participants hold both anticipated outcomes in working memory, and the first reward is thereby associated with both outcomes. Additionally, it is possible that during the second outcome participants infer the identity of the first outcome, which is then associated with the second reward. Thus we added two novel, MBCA free parameters $c_{RI,\;1\to 2}^{MB}$, $c_{RI,\;2\to 1}^{MB}$ where $c_{RI,\;i\to j}^{MB}\;$governs the extent to which on RI trials, the temporally i’th reward reinforces the MB values of the other (j’th) outcome. Accordingly, for the RI trials alone, Equation 5 was replaced with the following update (for all i = 1,2; j = 3-i):

(Equation 10) $$Q^{\text{MB}}\left(\text{outcome i}\right)\leftarrow \;\left(1-f^{MB}\right)*Q^{\text{MB}}\left(\text{outcome i}\right)+\;c_{RI,\;i}^{MB}*r_{outcome\;i}+c_{RI,\;j\to i}^{MB}*r_{outcome\;j}\;$$

Note that our full model, is nested under the “RI cross CA” (setting $c_{RI,\;1\to 2}^{MB}=c_{RI,\;2\to 1}^{MB}=0$).

#### Model fitting and model comparison

We fit our choice models to the data of each individual, maximizing the likelihood (ML) of their choices (we optimized likelihood using MATLAB’s ‘fmincon’, with 200 random starting points per participant; Table S1 for best-fitting parameters). Our full hybrid agents, which allowed for contributions from both an MB and an MF system, served as a super-model in a family of seven nested sub-models: 1) a pure MBCA agent, eliminated MFCA by constraining all MF parameters to 0: $c^{MF}=f^{MF}=0$, 2) a pure MFCA agent, eliminated MBCA by constraining all MB parameters to 0: $\forall format,\;pos:\;c_{format,\;pos}^{MB}=f^{MB}=0.$, 3) a ‘no presentation effects on MBCA’ sub-model which constrained equality across positions and formats, $\forall format,\;pos:\;c_{format,\;pos}^{MB}\equiv c^{MB}$, 4) a ‘No position effect on MBCA’ sub-model which forced for each format equality between the MBCA parameters for the first and second outcome $\forall format,\;c_{format,\;1}^{MB}=\;c_{format,\;2}^{MB}$; 5) a ‘No format effect on MBCA’ sub-model which constrained the MBCA parameters for all three format to be equal for each outcome position $\forall pos,\;c_{S,\;pos}^{MB}=\;c_{PI,\;pos}^{MB}=\;c_{RI,\;pos}^{MB}$; 6) an ‘additive-MBCA effects’ sub-model, which allowed format and position to exert additive effects on MBCA but not to interact $\forall format,\;pos,\;c_{format,\;pos}^{MB}\equiv \;c_{format}^{MB}+\;c_{pos=2}^{MB}\text{∗}1_{\text{pos}=2}$; and 7) a ‘flipped functional position’ sub-model which forced the following two constraints: $\;c_{S,\;1}^{MB}=\;c_{PI,\;1}^{MB}=c_{RI,2}^{MB}\equiv \;c_{1}^{MB}$ and $\;c_{S,\;2}^{MB}=\;c_{PI,\;2}^{MB}=c_{RI,1}^{MB}\equiv \;c_{2}^{MB}$. Note this last sub-model included only two free MBCA parameters. This model was designed to test an assumption that MBCA depends only on an outcome’s “functional position” rather than its temporal position. The functional position of an outcome reflects the order in which MBCA operates. Note that whereas for S and PI trials the temporal and functional order are equivalent (credit is first assigned to the temporally first outcome), for RI trials these orders are flipped such that the temporally first outcome is functionally second and vice versa.

We next conducted a bootstrapped generalized likelihood ratio test (BGLRT^52^) for the super-model versus each of the sub-models separately (Figures S4 and S7C). In a nutshell, this method is based on the classical-statistics hypothesis testing approach and specifically on the generalized-likelihood ratio test (GLRT). However, whereas GLRT assumes asymptotic Chi-square null distribution for the log-likelihood improvement of a super model over a sub-model, in BGLRT these distributions are derived empirically based on a parametric bootstrap method. In each of our model comparison the sub model serves as the H0 null hypothesis whereas the full model as the alternative H1 hypothesis.

For each participant, we created 1001 synthetic experimental sessions by simulating the sub-agent with the ML parameters on novel trial sequences which were generated as in the actual data. We next fitted both the super-agent and the sub-agent to each synthetic dataset and calculated the improvement in twice the logarithm of the likelihood for the full model. For each participant, these 1001 likelihood-improvement values served as a null distribution to reject the sub-model. The p value for each participant was calculated based on the proportion of synthetic dataset for which the twice logarithm of the likelihood-improvement was at least as large as the empirical improvement. Additionally, we performed the model comparison at the group level. We repeated the following 10,000 times. For each participant we chose randomly, and uniformly, one of his/her 1,000 synthetic twice log-likelihood super-model improvements and we summed across participant. These 10,000 obtained values constitute the distribution of group super-model likelihood improvement under the null hypothesis that a sub-model imposes. We then calculated the p value for rejecting the sub-agent at the group level as the proportion of synthetic datasets for which the super-agent twice logarithm of the likelihood improvement was larger or equal to the empirical improvement in super-model, summed across participants.

#### Model recovery

Because our model-comparisons are based on BGLRT, model recovery questions are tantamount to assessing type-I error rates and power. We used a type I error rate of 0.05 meaning that by design, if a null model (one of our sub-models) generated the data it will not be rejected (i.e., it will be “recovered”) with a probability of 0.95. To estimate the power of our design, we repeated the same steps as in our BGLRT analysis, but this time using synthetic data that was simulated from the full model instead of the sub-models. For each simulated dataset, we examined whether each of the null models were rejected, according to BGLRT, in favor of the full model. We found that for all 10,000 simulations, all sub models were rejected in favor of the full model (i.e., the full model was “recovered”) at the group level. Thus the estimated power is very close to 1 for assessing all sub-models.

#### Model simulations

To generate model predictions with respect to choices, we simulated for each participant, 25 synthetic experimental sessions (novel trial sequences were generated as in the actual experiment), based on his or her ML parameters obtained from the corresponding model fits (the models are described above). We then analyzed these data in the same way as the original empirical data (but with datasets that were 25 times larger, as compared to the empirical data, per participant).

#### Analysis of model parameters

For each participants we obtained, based on the full model, six MBCA parameter estimates corresponding to format x position. We then ran a mixed effects model (implemented with MATLAB’s function “fitglme”) for the CA parameters (denoted C), with participants (PART) serving as random effects with a free covariance matrix. Two regressors PROSPECTIVE, and RETROSPECTIVE, indicated whether format was prospective-inference or retrospective-inference, respectively, and the regressor POSITION, coded the serial position of the common outcome on trial n (0.5: first, −0.5: second). The model, in Wilkinson notation, was: CA ∼(PROSPECTIVE + RETROSPECTIVE)^∗^POSITON + (PROSPECTIVE + RETROSPECTIVE+ POSITON |PART). Contrasts were tested in a similar way to that described in Model-agnostic analysis: MFCA and MBCA.

We also ran a similar model but regressing FUNCTIONAL-CA instead of CA. This variable was identical to CA after flipping the order of the 2 MBCA parameters in the RI position. Thus, in this model the regressor POSITION captures the effect of the functional-serial position, under the assumption that the functional and temporal positions are flipped in RI. Finally, the two MBCA parameters of the “flipped functional position” sub-model were compared using a t test.

#### Parameter recovery and trade-offs

We tested parameter recovery of our full model (Figure S5) based on the following method. For each participant, we create 1,000 synthetic datasets which were same in size and structure to the empirical sessions, by simulating the full model based on his/her best fitting parameters. We then fitted these datasets with the full model. We assessed the Pearson correlations between each generating MBCA parameter and the corresponding recovered parameter across these 38^∗^1000 datasets.

Additionally, for each MBCA parameter and each synthetic dataset (of 38,000 datasets) we calculated the estimation error as the difference between the generating parameter and the recovered parameter. We assessed parameter trade-offs by calculating Pearson correlations between estimation errors for each pair of parameters.

#### Power of mixed-effects analyses

Prior to data-collection, we did not know what effect-size to expect (as far as we know, this is the first study to address the interaction between inference and different forms of CA). Thus, we collected a sample of comparable size to our previous tasks.^14^^,^^16^^,^^19^ However, an examination of the power of the high order interactions in our mixed effects analyses can guide future studies. We addressed this question by taking 1000 bootstrap samples of our participants and ran model agnostic analyses of interest for each bootstrap sample, examining focal high-order interaction effects. The power to detect an interaction was estimated as the proportion of bootstrap samples for which an interaction was significant at p < 0.05. For our main model-agnostic analysis (Figure 2E) the estimated power for the triple common-reward x format x position interaction was 0.57. For the mixed effects model based on estimated model parameters (Figure 3A) the estimated power for the format x position interaction was higher, 0.68. Thus, we recommend using larger samples in future studies. For example, when rerunning our power simulations for “up-sampled” cohorts of 60 participants, we found high power for both model agnostic (.84) and the parameter-based (.87) analyses.

#### Simulations of reward-earnings

We assessed the instrumental value of the MBCA parameters our participants used in each format for acquisition of rewards (Figure 5A). We yoked each real participant with 3 synthetic pure-MBCA agents: S, PI and RI. For agent S we duplicated the participant’s MBCA parameters from the standard format across all 3 formats: $\forall format,\;pos,\;sc_{format,\;pos}^{MB}=\;c_{S,\;pos}^{MB}$, where sc denoted the MBCA parameters for a synthetic agent. PI and SI agents were defined similar based on the real participant’s PI and RI parameters, respectively. For synthetic agents, $f^{MB}$ was set equal to the empirical forgetting rate of the true participant and MFCA and perseveration were ablated: $c^{MF}=f^{MF}=f^{P}=pr=0$.

Each synthetic agent was tested on the same 1000 synthetic experimental sessions that were constructed in the same way as the empirical sessions. For each agent, we calculated, the average number of points it earned per-trial based on its simulated choices and consequent rewards. We compared these average earnings to those on the same experimental trials of two additional hypothetical agents: ‘guessing’ and ‘oracle’. On each trial, the guessing agent earned the average expected total reward (according to the generating outcome-reward probabilities) provided by both bandits, whereas the oracle agent omnisciently earned the maximal total expected reward across the two offered bandits. For comparison, we calculated the standardized earnings of the synthetic MBCA agent as the ratio of how much more it earned than the guessing agent to how much more the oracle agent earned than the guessing agent. Finally, for each experimental session we averaged standardized earnings across each group of synthetic agents. We thus obtained 1000 repeated-measurements of group-level standardized earning for S, PI, and RI agents. For each agent we averaged these 1000 measurements (Figure 5A).

We assessed how earning change as function of a trade-off between MBCA for the seen and hidden outcomes in the retrospective inference format (Figure 5B). We simulated 10,000 pure MBCA-agents for each of several combinations of MBCA parameters on synthetic experimental sessions and calculated standardized earnings (as above). In all simulations the MB forgetting rates was set to the estimated empirical group-level average and agents expressed no choice-perseveration tendencies or MFCA: $c^{MF}=f^{MF}=f^{P}=pr=0$. The parameter combinations we used is the various simulations are as follows. We defined a grid of total MBCA ranging from 0.3 to 3 in steps of 0.3, and a percentage-grid from 0 to 1 in steps of 0.05. For each level of total MBCA, t, and percentage, p, we included the parameter combination:

$$\;c_{S,\;1}^{MB}=\;c_{S,\;2}^{MB}=\;c_{PI,\;1}^{MB}=\;c_{PI,\;2}^{MB}=\frac{t}{2};\;c_{RI,\;1}^{MB}=pt,\;c_{RI,\;1}^{MB}=\left(1-p\right)t\;$$

## Supplemental information

Document S1. Figures S1–S7 and Table S1

Document S2. Article plus supplemental information
