# Supplementary material for: Efficiency and prioritization of inference-based credit assignment
Source: Curr Biol. 2021 Jul 12;31(13):2747–2756.e6. doi: 10.1016/j.cub.2021.03.091 (PMC8279739; doi:10.1016/j.cub.2021.03.091)
Supplement: Document S1. Figures S1–S7 and Table S1 [file mmc1.pdf]

Current Biology, Volume *31*

Supplemental Information

Efficiency and prioritization of inference-based credit assignment

Rani Moran, Peter Dayan, and Raymond J. Dolan

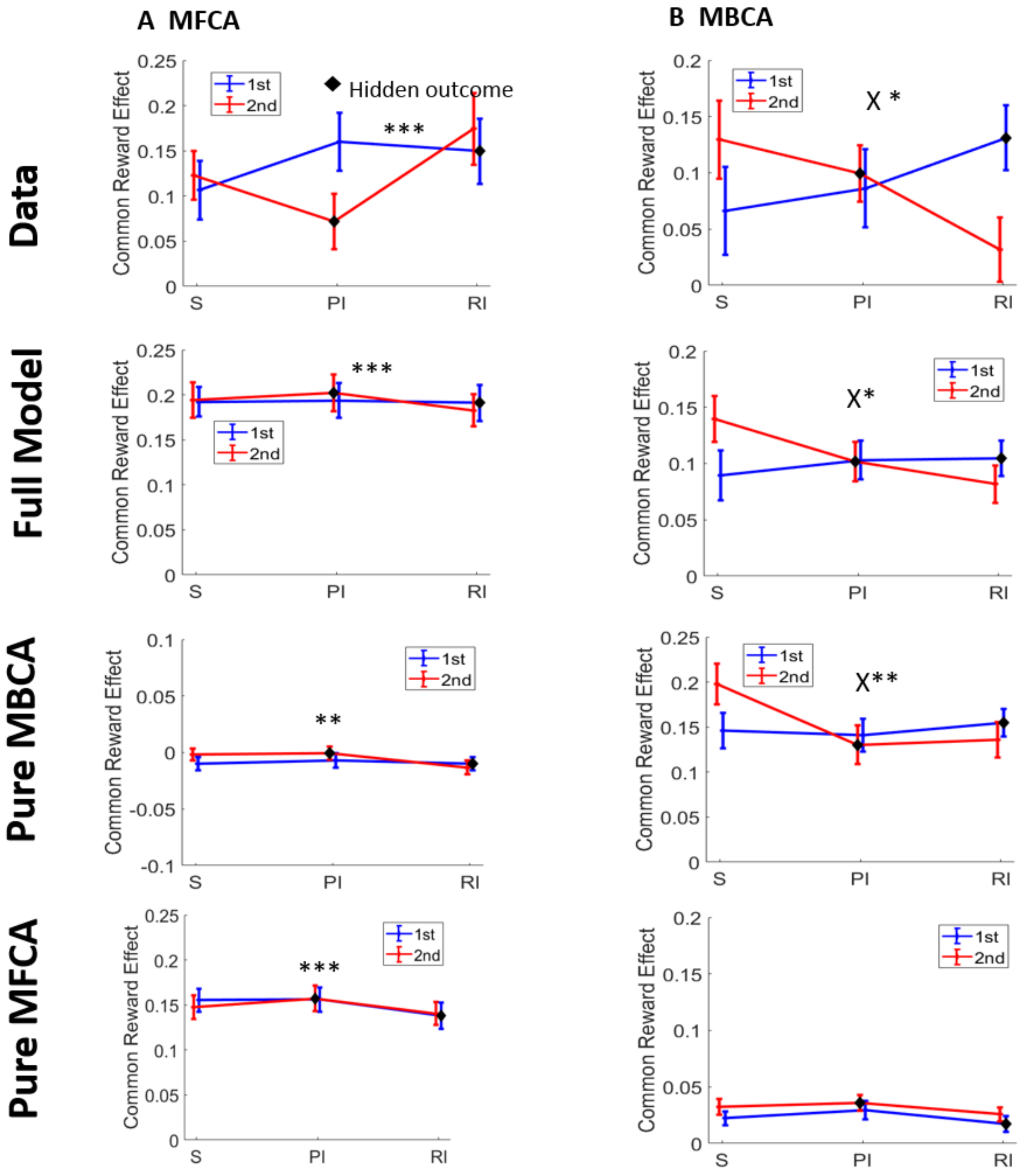

Figure S1. Signatures of MBCA and MFCA. **Related to Figure 2.** A) The Data panel is Figure 2C (empirical effects on choice repetition) and the Full-model panel is Figure 3B (both copied for convenience). The other panels correspond to the same analysis for the non-hybrid, “pure”, sub-models. The pure MFCA sub-model captured the positive common-reward main effect on choice-repetition but the pure MBCA sub-model did not, predicting a tiny negative effect (note the different scale for this panel) B) Similar to (A) but for the analysis probing MBCA (Figures 2E, 3C). The pure MBCA sub-model captured the triple common-reward x format x position interaction on choice-generalization but the pure MFCA did not. Error bars correspond to SEM across participants calculated separately in each condition (n=38). *,**,*** denote p<.05, .01, .001, respectively. p-values were calculated based on mixed effects logistic regression models.

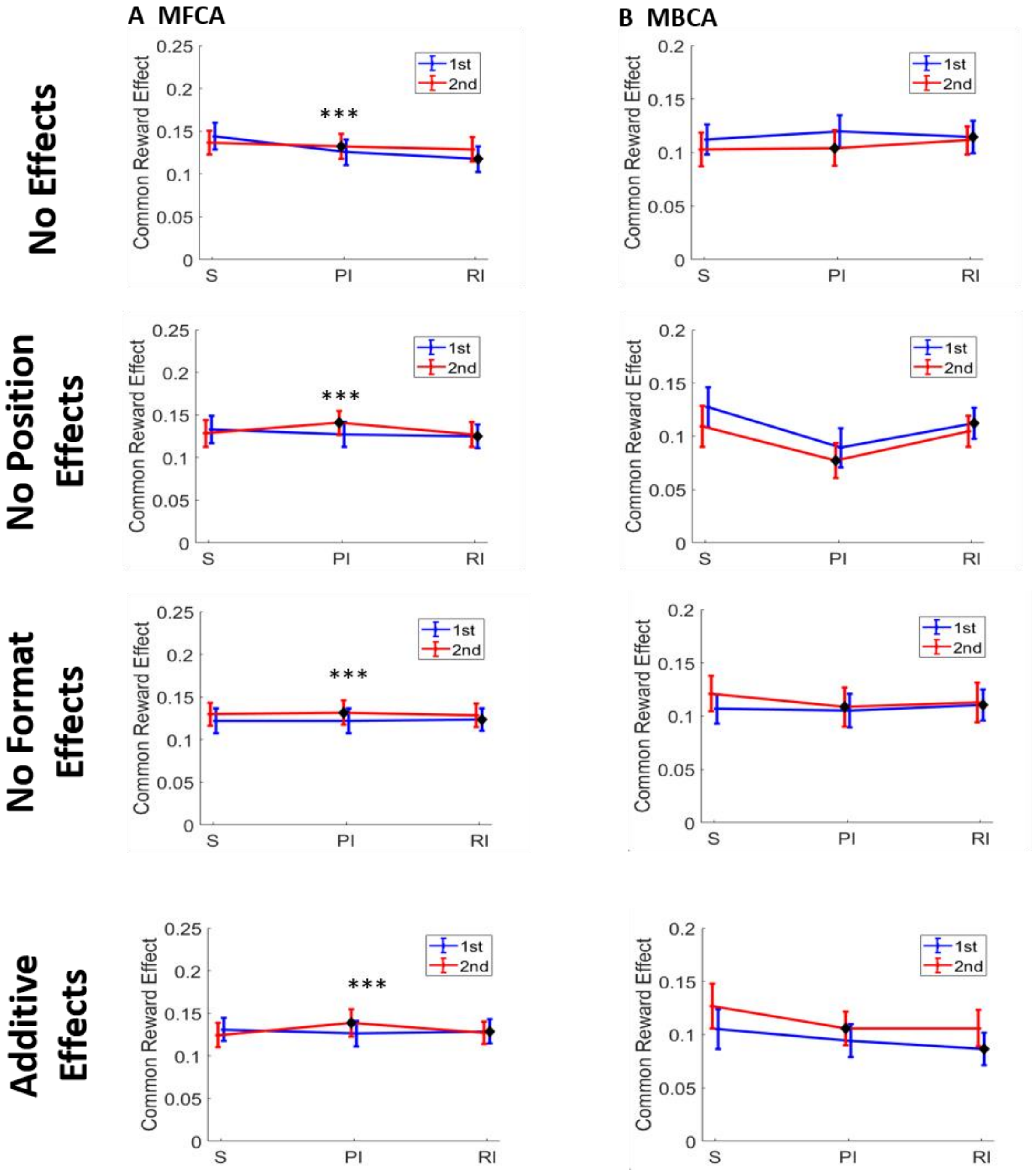

Figure S2. Signatures of MBCA and MFCA. Related to Figure 2. The arrangement of this figure similar Figure S2. Here simulation results are shown for the hybrid sub-models which constrained the flexibility of MBCA parameterisation. A) All sub-models captured the common-reward main effect on choice repetition. B) None of the models captured the triple Common-reward x format x position interaction on choice-generalization. Error bars correspond to SEM across participants calculated separately in each condition (n=38). *** denotes p<.001. p-values were calculated based on mixed effects logistic regression models.

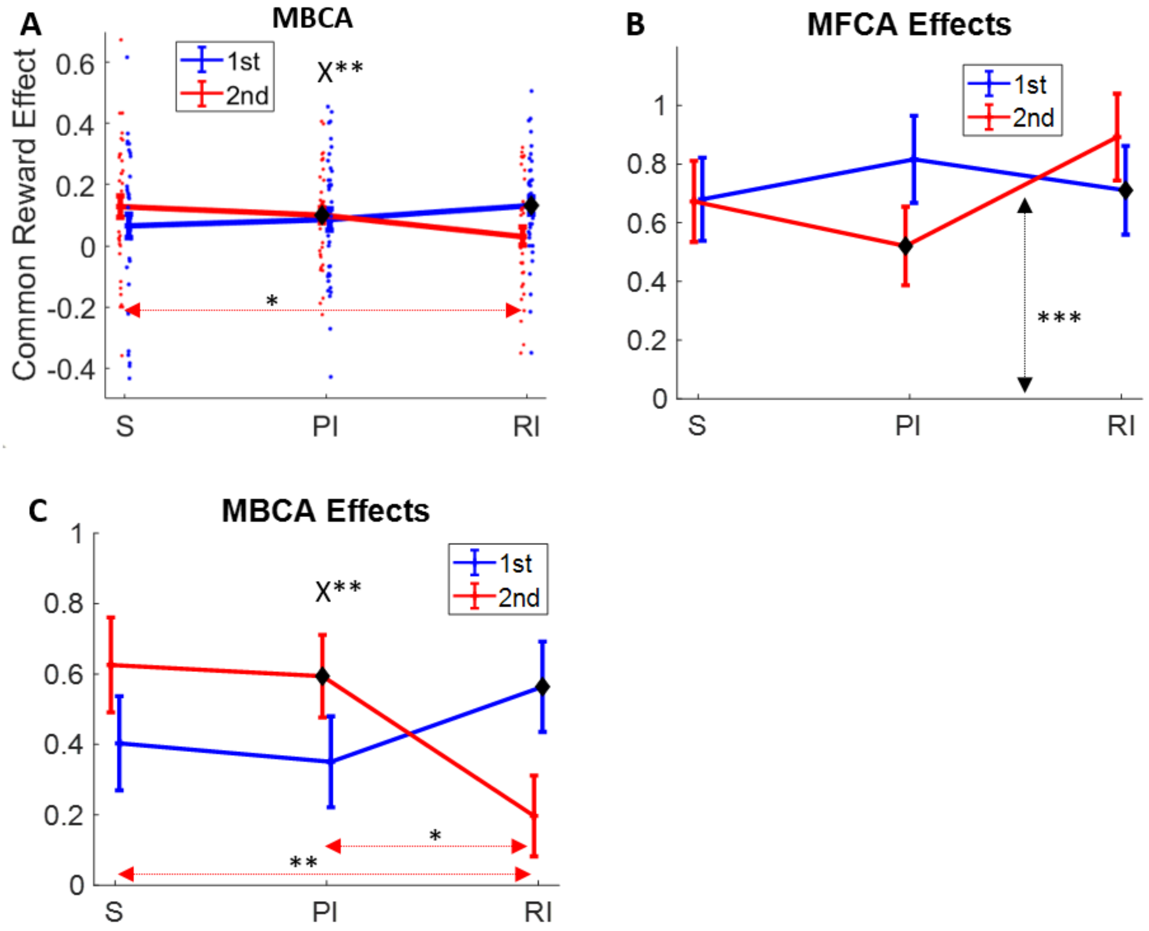

Figure S3. Additional 'model agnostic analysis'. Related to Figure 2. A) Same as Figure 2E but individual-participant data is also shown (dots). We conducted further analyses, to probe deeper into MBCA effects, wherein we regressed, using a mixed effects model, the reward effects (i.e., the difference between repetition probabilities following a common reward and non-reward; displayed) on format and serial position (Methods). Neither main effects for position (comparing averages along curves) nor format (comparing averages across curves) reached significance (both p>.763) but the format x position interaction (F(2,222)= 4.97, p=.008) was significant. Specifically, the retrospective inference format interacted with position in a manner that was different to the standard format (b=.16, t(222)= 3.1, p= .002; cross over in curves between RI and S; the other two format pairs didn't interact differently with position, both p> .055). Interpreting this further, whereas for the first outcome there were no differences between the retrospective inference and the standard format (b= .07, F(1,222)= 2.73, p=.1; for the 1st position curve S and RI are on a par) for the second outcome, the reward effect was smaller in the retrospective than the standard format (b=-.1, F(1,222)= 6.62, p=.011; for the 2nd position curve RI<S). These findings converge to provide the same conclusions as the analysis in the main text. B-C) MFCA and MBCA effects as a function of format and serial position based on our model-agnostic mixed effects model that included all trial transitions. One benefit of our model-agnostic analyses, described in the main text, is they provide intuitive and relatively simple signatures that dissociate MFCA and MBCA contributions. However, these analyses are based on different subsets of trial transitions (Figures 2B, D) and do not take account of the entire set of CA influences from a previous trial. For example, our MFCA analysis (Figures 2B-C) controlled neither for MFCA nor

MBCA influences that a trial-n non-common reward (i.e., reward to the outcome not designated as common; Garlic in the current example) exerts on choice repetition (on trial n+1). To address these potential limitations, we conducted a further model agnostic analysis that included all trial transitions and modelled simultaneously the entire set of putative MFCA and MBCA influences (STAR Methods). For MFCA (panel B), we found a positive main effect for reward ($b=0.72$, $F(1,12781)= 61.77$, $p=4e-15$). However, there was no evidence that reward interacted with either format, position of both (all $p>.085$). The asterisk corresponds to the significance of the MFCA effect, the average of all data points (black line). For MBCA (panel C), we found a positive main effect of reward ($b=0.46$, $F(1,12781)= 45.86$, $p=1e-11$). There was no evidence reward interacted with either format or position (both $p>.486$). However, we found a triple reward*position*format interaction ($F(2,12781)= 5.05$, $p=.006$). Specifically, position modulated the reward effect differently in the standard compared to RI format ($b=-0.59$, $t(12781)= -2.71$, $p=.007$) and in PI compared to RI ($b=-0.61$, $F(1,12781)= 7.22$, $p=.007$), but there was no evidence that position modulated MBCA differently between the S and PI formats ($p=.93$). Interpreting these interactions further we found that for the first outcome, the reward effect didn’t differ between the RI format and either the PI or S formats (both $p>.173$). In contrast, for the second outcome, the reward effect in the RI format was lower than both S ($b=-0.43$, $F(1,12781)= 7.09$, $p=.008$) and PI ($b=-0.40$, $F(1,12781)= 6.02$, $p=.014$). The asterisks correspond to the significance of the triple interaction (see X; reward x format x position) and to the contrasts between the reward effect in RI and S, and RI and PI for the second outcome (red lines). Error bars correspond to SEM across participants calculated separately in each format. *,*** denote<.05, .001, respectively. P-values were calculated based on mixed effects logistic regression models. Black diamonds designate concealed outcomes.

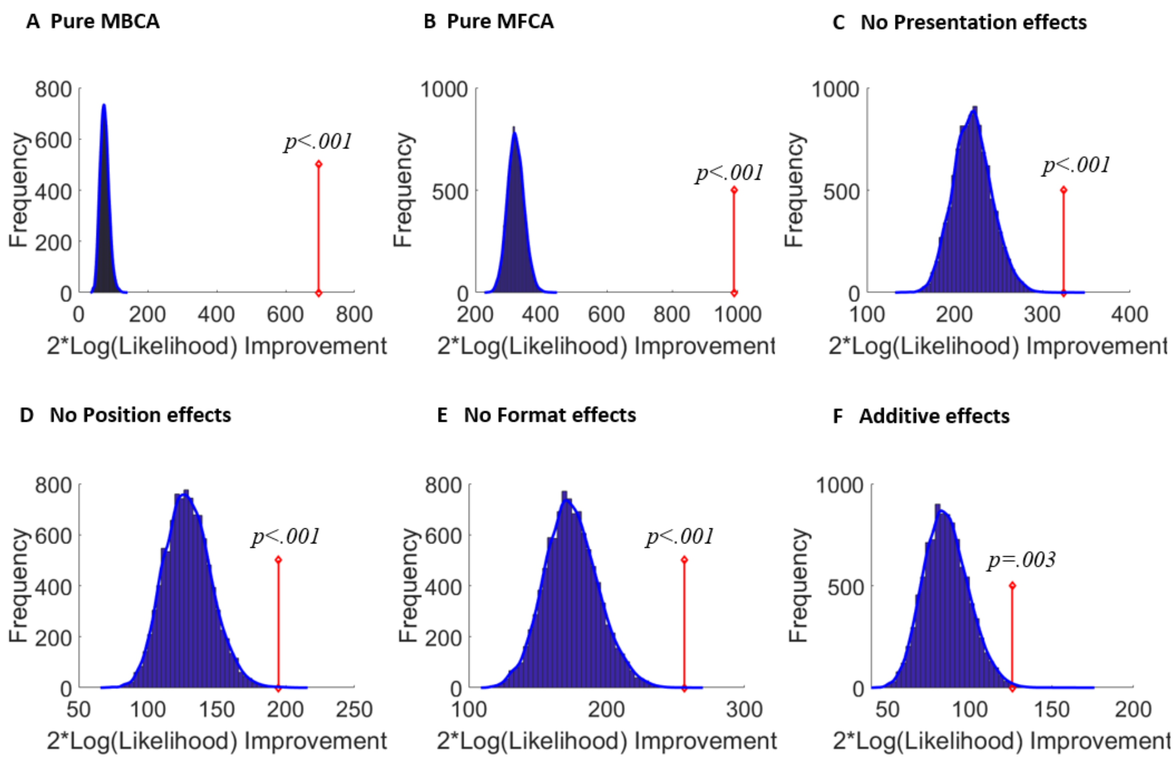

Figure S4. Model-comparison results. Related to Figure 3. A) Results of the bootstrap-GLRT model-comparison for the pure MBCA sub-model. The blue bars show the histogram of the group twice log-likelihood improvement (model vs. sub-model) for synthetic data simulated using the sub-model (10,000 simulations). The blue line displays the smoothed null distribution (using Matlab's "ksdensity"). The red line shows the empirical group twice log-likelihood improvement. 0 out of the 10000 simulations yielded an improvement in likelihood that was at least as large as the empirical improvement. Thus, the sub-model can be rejected with p<.001. B-E) Same as (A), but for the pure MFCA (p<.001), the no presentation effect on MBCA (p<.001), the no position effect on MBCA (p<.001), the no format effect on MBCA and the additive MBCA effects MBCA (p=.003) sub models. The various models, the model-comparison method and model-recovery performance are detailed in the STAR Methods.

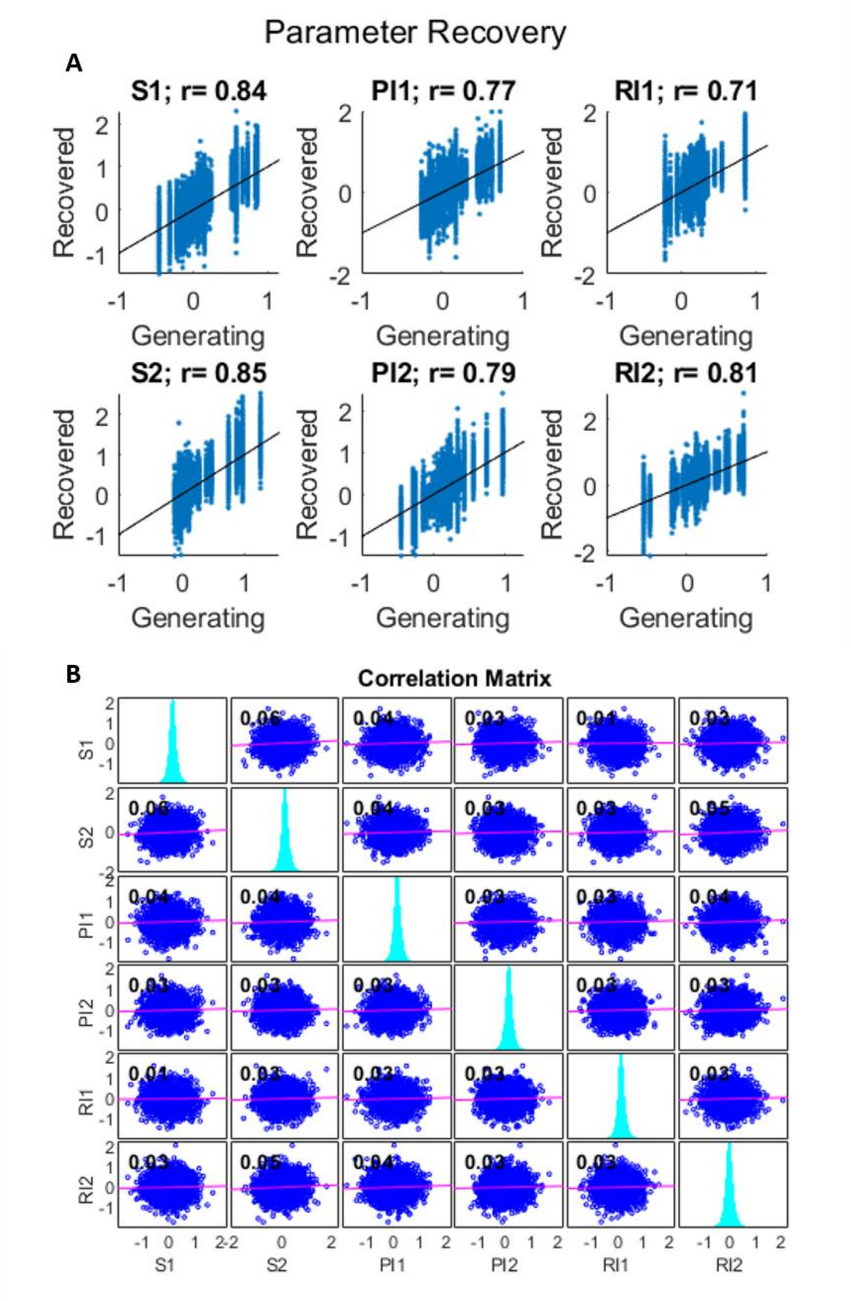

Figure S5. Parameter recovery and trade-off for the full model. Related to Figure 3. A) Each panel displays a scatter plot of a generating MBCA parameter (abscissa) vs. the recovered parameters (ordinate; STAR Methods). Black solid lines are imposed diagonals. Model recovery for MBCA parameters was high [mean Pearson's r = .79]. B) Each non-diagonal panel displays a scatter plot between estimation errors (recovered minus generated parameters) for a pair of MBCA parameters. The numbers in the top left are Pearson's r and regression lines are imposed. Diagonal panels display estimation-error distributions for the various MBCA parameters. Trade-offs are negligible (all |r|<.064). S=Standard, PI=prospective inference, RI=retrospective inference; The 1/ 2 in panel titles refer to the outcome's serial position.

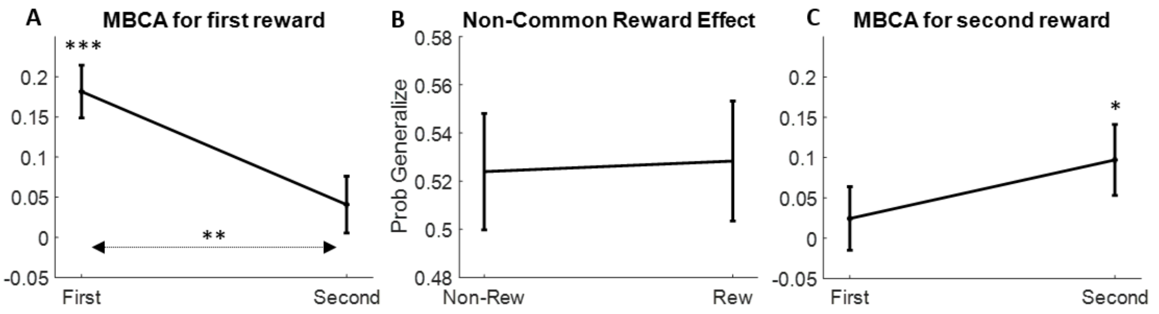

Figure S6. Examining whether Cross CA occurs in the RI format. Related to Figure 6. A) As confirmation to the results presented in Figure 6B, we also fitted to our data a flexible model (dubbed the "RI cross-CA" model; STAR Methods), wherein in the retrospective-inference format, each reward (first/second) was associated with two (instead of one) MBCA parameters, corresponding to the extents to which a reward reinforced 1) its own outcome, and 2) the other choice-related outcome. Examining the best fitting parameters of this model (displayed), we found that MBCA for the first outcome (based on its own reward) in the RI format was significantly positive ($M= 0.18$, $t(37)= 5.55$, $p=3e-6$) and larger than MBCA for the second outcome (based on the first reward; $t(37)= 2.75$, $p=.009$), which was non-significant ($M= 0.04$, $t(37)= 1.15$, $p=.256$). The upshot is that credit from the first reward is selectively assigned to the first outcome with no evidence it spills to the second outcome. B) For completeness, we tested whether in the RI format cross-CA occurs from the second reward spills to the first outcome. Same as Figure 6B but here we analysed only choice-generalization trials wherein the common outcome was first. Regressing choice-generalization on the non-common reward using a mixed-effects logistic model (Methods) revealed no significant effect ($b= 0.02$; $t(74)= 0.14$, $p= .89$). C) Same as A but examining MBCA for the second reward. The MBCA parameters for the second reward was significantly positive for the second outcome alone (second outcome: $M=0.1$, $t(37)= 2.2$, $p=.034$; first outcome: $M=0.02$, $t(37)= 0.62$, $p=.54$). Here, the contrast was non-significant ($t(37)=-1.16$, $p= .255$), likely due to the lower levels of self-MBCA for this outcome. Hence, there is no evidence for cross-CA for the second outcome. Error bars correspond to SEM across participants calculated separately in each condition ($n=38$). *,**,*** denotes $p<.05$, .01, .001, respectively.

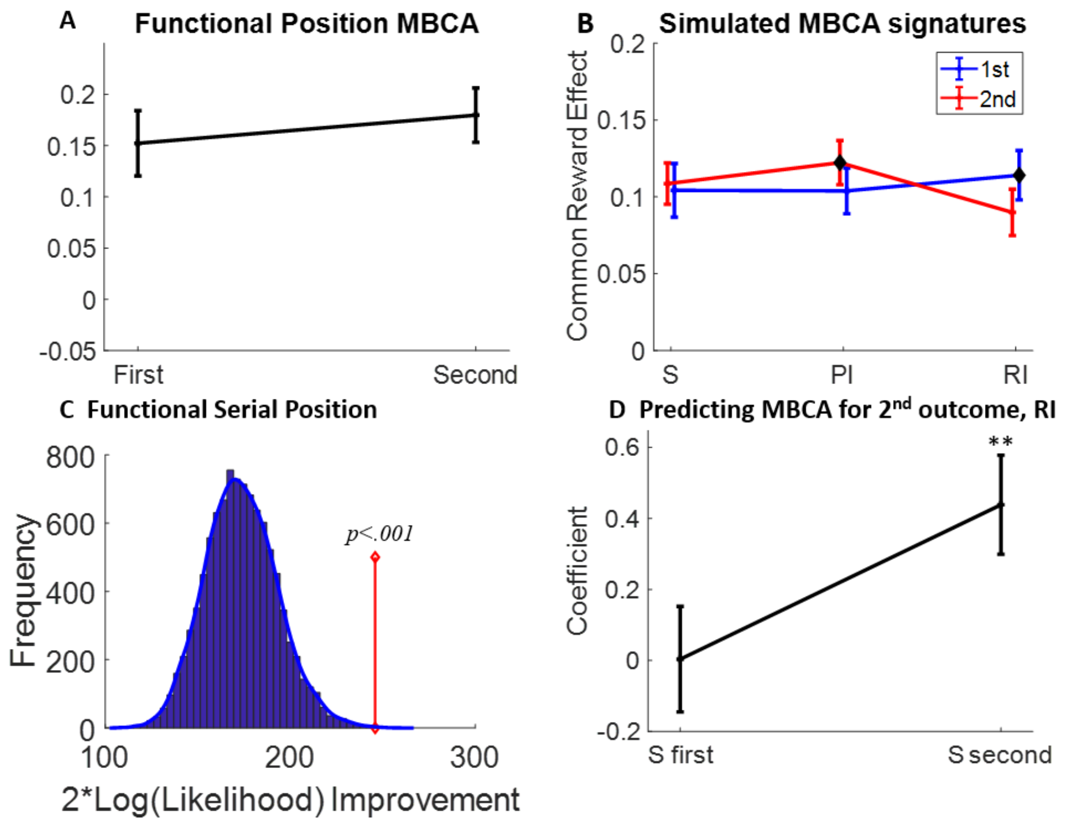

Figure S7. Examining the possibility of a functional, rather than temporal, serial position effect. Related to Figure 6. A third alternative account for our findings is that the sole effect of the retrospective-inference format could be to flip the 'functional-order' of CA between the two outcomes during feedback (i.e., as the second outcome appears, people first assign credit to it and next infer and assign credit to the first outcome). According to this account, our findings might simply reflect a functional, rather than a temporal, serial-order effect such that MBCA is stronger for the functionally second outcome. Note that in the standard and prospective-inference formats the functional and the temporal serial orders are identical. We addressed the latter possibility by formulating an additional 'flipped functional order' sub-model in which we had two MBCA parameters, one for each functional (rather than temporal) position (STAR Methods). A) Examining the best fitting parameters for this sub-model (displayed), we found no significant difference between these two MBCA parameters ($t(37)= 1$, $p=.325$). Relatedly, when we re-ran our mixed effects model on the parameters of the full model but including functional, instead of temporal, serial position as a regressor, we found no functional-position effect ($p=.104$). Thus, we found no evidence MBCA is stronger for the functionally second position. B) Simulating this sub-model, failed to predict the significant focal common-reward x position x format interaction pertaining to MBCA signatures (compare to Figure 2E). C) a bootstrapped generalized likelihood ratio test rejected the flipped-functional order sub-model at the group level ($p<.001$) in favour of the full model. D) based on the full model, we regressed the MBCA parameter for the seen (temporally second) outcome in the RI format on both MBCA parameters in the standard format, allowing these two parameters to compete for accounted variance. The coefficient of the MBCA parameters for the second S-outcome alone was significant (First outcome: $b=0$, $t(35)=0.02$, $p=.982$; Second outcome: $b=0.44$, $t(35)=3.14$, $p=.003$). One would expect the opposite pattern had the decline in MBCA for the second seen RI outcome (relative to

the second standard outcome) occurred simply because its functional position was first. Taken together, our findings speak against the possibility that flipped functional positions in RI is the sole explanation to our findings. Error bars correspond to SEM across participants calculated separately in each condition (n=38). ** denotes p<.01.

Param	$c_{S,1}^{MB}$	$c_{S,2}^{MB}$	$c_{PI,1}^{MB}$	$c_{PI,2}^{MB}$	$c_{RI,1}^{MB}$	$c_{RI,2}^{MB}$	c^{MF}	pr	f^{MF}	f^{MB}	f^{P}
Mean	0.16	0.25	0.15	0.15	0.16	0.10	0.70	0.03	.29	.16	.37
(SE)	(0.09)	(0.10)	(0.07)	(0.08)	(0.06)	(0.08)	(0.14)	(0.13)	(.08)	(.09)	(.10)

Table S1. Best fitting parameters for the full model. Related to Figure 3. See 'Computational Models' in STAR Methods for a full description of the model and its parameters.
